# Listeria in Food: Prevalence and Control

**DOI:** 10.3390/foods12071378

**Published:** 2023-03-24

**Authors:** Araceli Bolívar, Fernando Pérez-Rodríguez

**Affiliations:** Department of Food Science and Technology, UIC Zoonosis y Enfermedades Emergentes ENZOEM, ceiA3, Universidad de Córdoba, 14014 Córdoba, Spain; b42perof@uco.es

*Listeria monocytogenes* is a foodborne pathogen characterized by its psychrotrophic and ubiquitous nature as well as its ability to survive and proliferate in a wide range of harsh environments and foods. These features make the pathogen a primary concern in the food industry, especially in the cold chain of ready-to-eat (RTE) food products. Although the incidence of listeriosis is low compared to other foodborne illnesses (e.g., salmonellosis), its high hospitalization and case fatality rates, mainly in high-risk population groups, pose a significant threat to public health.

The prevalence of *L. monocytogenes* has been reduced in many food categories over the last two decades, especially in meat and meat products, due to the application of improved control measures. As highlighted by Abdeen et al. [1] in this Special Issue, the application of suitable control measures along the food chain to reduce pathogen levels and prevent product recontamination together with the continuous training of food handlers are key to reduce the pathogen incidence. They found that the prevalence of *L. monocytogenes* in different RTE food products from Egypt was higher compared to other *Listeria* species. In addition, the pathogen isolates carried multiple virulence-related genes (*hlyA*, *iap*, and *actA*) and showed phenotypic resistance to six antibiotics. This highlights the importance of monitoring the emergence of resistant and virulent strains.

The presence of persister cells and biofilms in food processing environments also requires attention. In the study by Panebianco et al. [2], the effectiveness of gaseous ozone against the biofilm of *L. monocytogenes* isolates from different sources was evaluated. They concluded that ozone gas was not sufficient to completely counteract the pathogen biofilm, but it may be useful as an additional tool to improve the existing sanitization procedures in food processing environments. On the other hand, the development of innovative control approaches with reduced environmental impact is necessary to offer consumers more natural solutions and chemical-free products. In this context, van Gijtenbeek et al. [3] assessed the bioprotective potential of the *Lacticaseibacillus rhamnosus* strain Lrh-FQ to inhibit the growth of *L. monocytogenes* in creamed cottage cheese. The mechanism underlying the pathogen inhibition was based on competitive exclusion through the depletion of manganese content in the food matrix by Lrh-FQ.

A rapid and accurate detection of *L. monocytogenes* in food is also important to avoid sanitary and economic problems. Different methods are currently used for the detection of *L. monocytogenes* in food, such as conventional methods based on ISO standards using chromogenic media or biochemical tests. Alternative methods that reduce timing in pathogen detection are needed to prevent its dissemination through the food chain. In this context, Estévez et al. [4] evaluated the Vitek Immuno Diagnostic Assay System (VIDAS) to detect and count *L. monocytogenes* in various food items, demonstrating that VIDAS showed high efficiency and was not influenced by the food matrix or interfering microorganisms.

Predictive microbiology is a useful tool to estimate food shelf life and assist regulators in decision making. The development and/or validation of predictive models in real food products and the use of pathogen isolates from particular foods are essential to obtain accurate predictions of food systems. In this regard, Posada-Izquierdo et al. [5] modeled the effect of salt concentration on autochthonous isolated *L. monocytogenes* strains in an artisanal fresh cheese. Finally, Bolívar et al. [6] quantified and modeled the growth dynamics of six *L. monocytogenes* strains isolated from different fish products in salmon pâté. Both studies have demonstrated the growth potential of the pathogen under all tested conditions, providing interesting data about its kinetic behavior in RTE food products with significant consumption and commercial value.

The prevention of listeriosis relies on a comprehensive approach from farm to fork. This Special Issue of *Foods*, including five original articles and one short research communication, provides a deep understanding on the prevalence and genetic characteristics of *L. monocytogenes*, its growth dynamics in different RTE food products by suitable predictive tools, as well as the efficacy of different detection and control approaches. This insight can support the development of new and robust risk management strategies aimed at reducing the risk of listeriosis.
